# Supplementary material for: Impact of evolving greenhouse gas forcing on the warming signal in regional climate model experiments
Source: Nat Commun. 2018 Apr 3;9:1304. doi: 10.1038/s41467-018-03527-y (PMC5880811; doi:10.1038/s41467-018-03527-y)
Supplement: Supplementary file 1 — Supplementary Information [file 41467_2018_3527_MOESM1_ESM.pdf]

Supplementary Information

Impact of evolving vs. constant GHG forcing in regional climate modeling evidenced from the warming signal

S. Jerez*[1,2], J. M. López-Romero[1], M. Turco[3], P. Jiménez-Guerrero[1], R. Vautard[4] and J. P. Montávez[1]

[1] Regional Atmospheric Modeling Group, University of Murcia, 30100 Murcia, Spain

[2] Laboratrio associado IDL, Faculdade de Ciencias, Universidade de Lisboa, 1749-016 Lisboa, Portugal

[3] Department of Applied Physics, University of Barcelona, 08028 Barcelona, Spain

[4] Laboratoire des Sciences du Climat et de l'Environnement (LSCE), IPSL, CEA-CNRS-UVSQ, 91191 Gif sur Yvette, France

* Corresponding author: sonia.jerez@gmail.com

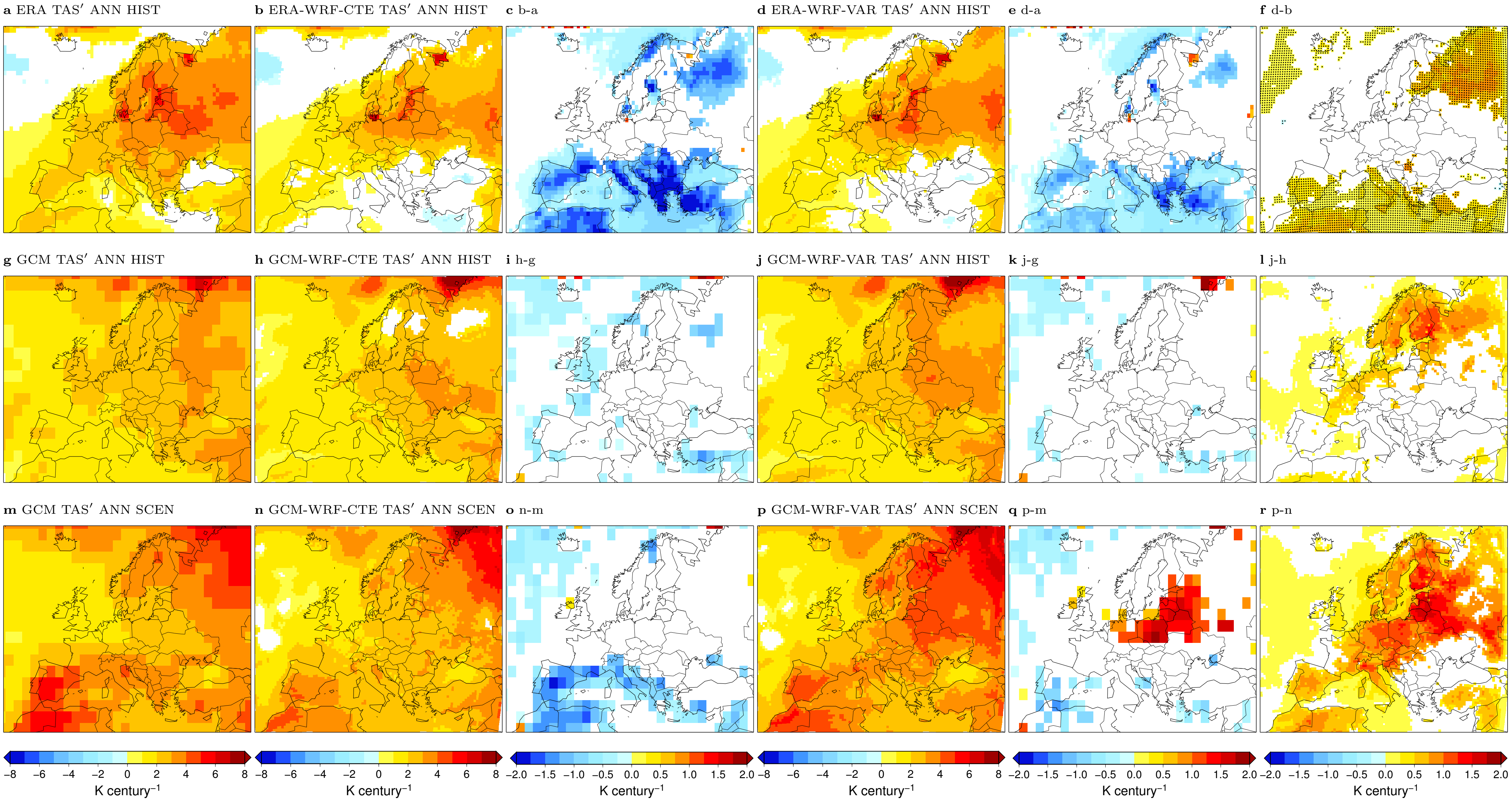

Supplementary Figure 1: Trends of the simulated yearly-mean time series of TAS in the historical period 1951-2005 (first and second rows) and the scenario period 2006-2050 (third row). The first row depicts the results from ERA (a) and ERA-driven WRF experiments (CTRL configuration, see Table 1 in the main manuscript), CTE (b) and VAR (d), along with the differences between ones and others: CTE minus ERA (c), VAR minus ERA (e) and VAR minus CTE (f). Similarly, the second and third rows depicts the results from GCM and GCM-driven WRF experiments. Only significant values ($p<0.1$) are shown. The points in the last column indicate that the magnitude of the difference between the VAR and CTE experiments is equal to or greater than the magnitude of the signal from the respective CTE experiment. Units: K century^{-1}. (Note: this is Figure 1 in the article.)

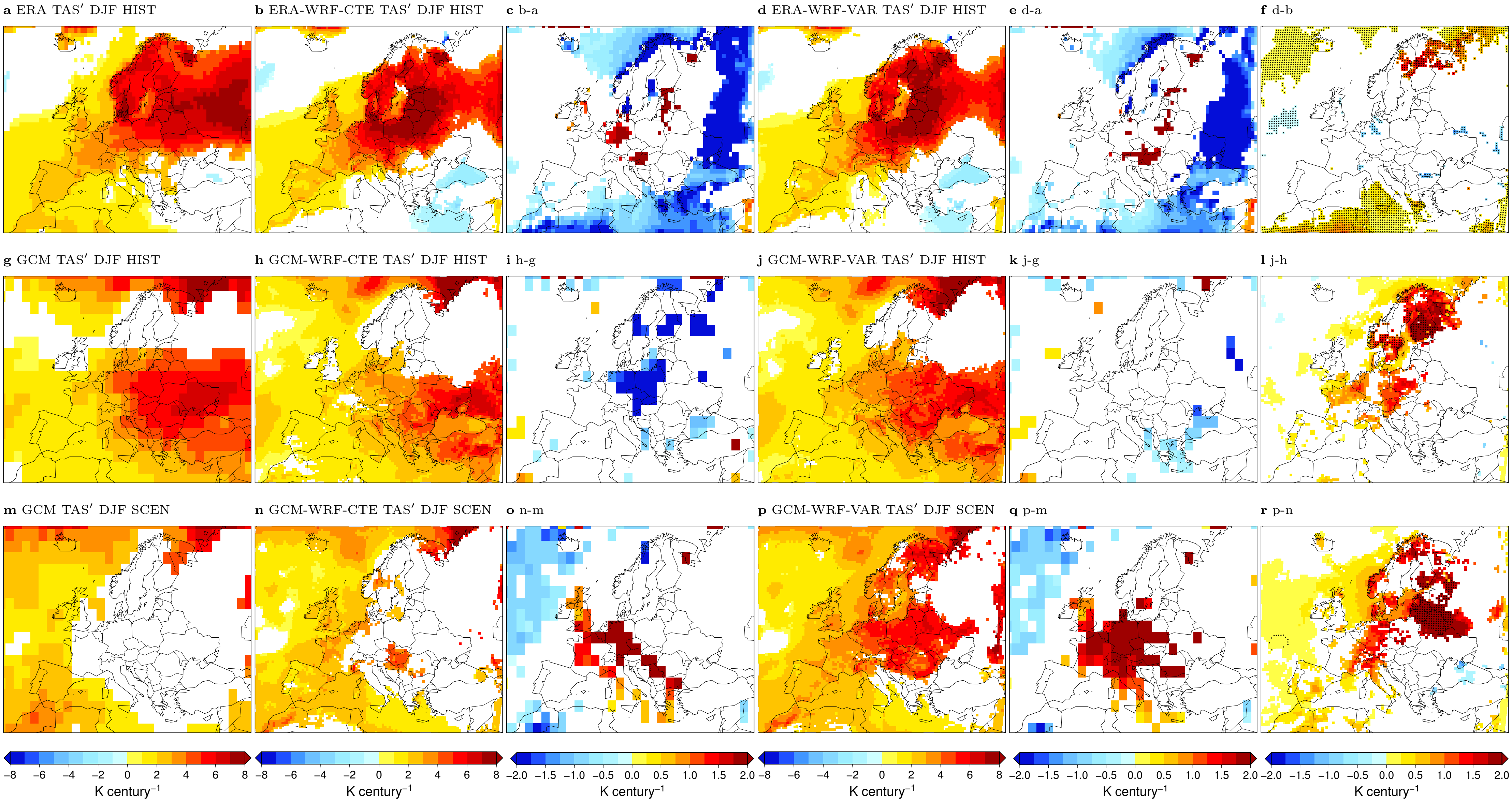

Supplementary Figure 2: As Supp. Fig. 1 for DJF-mean TAS.

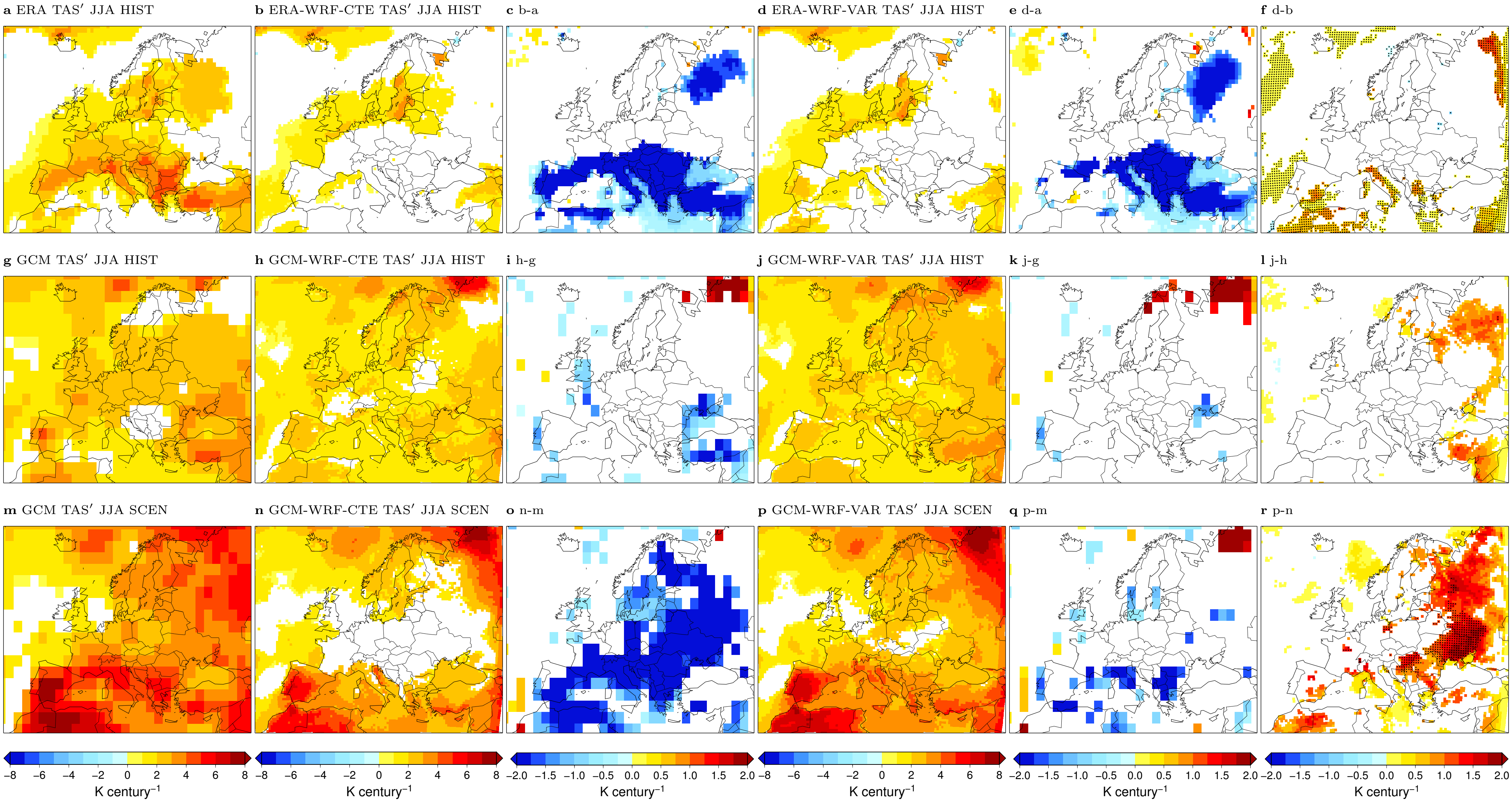

Supplementary Figure 3: As Supp. Fig. 1 for JJA-mean TAS.

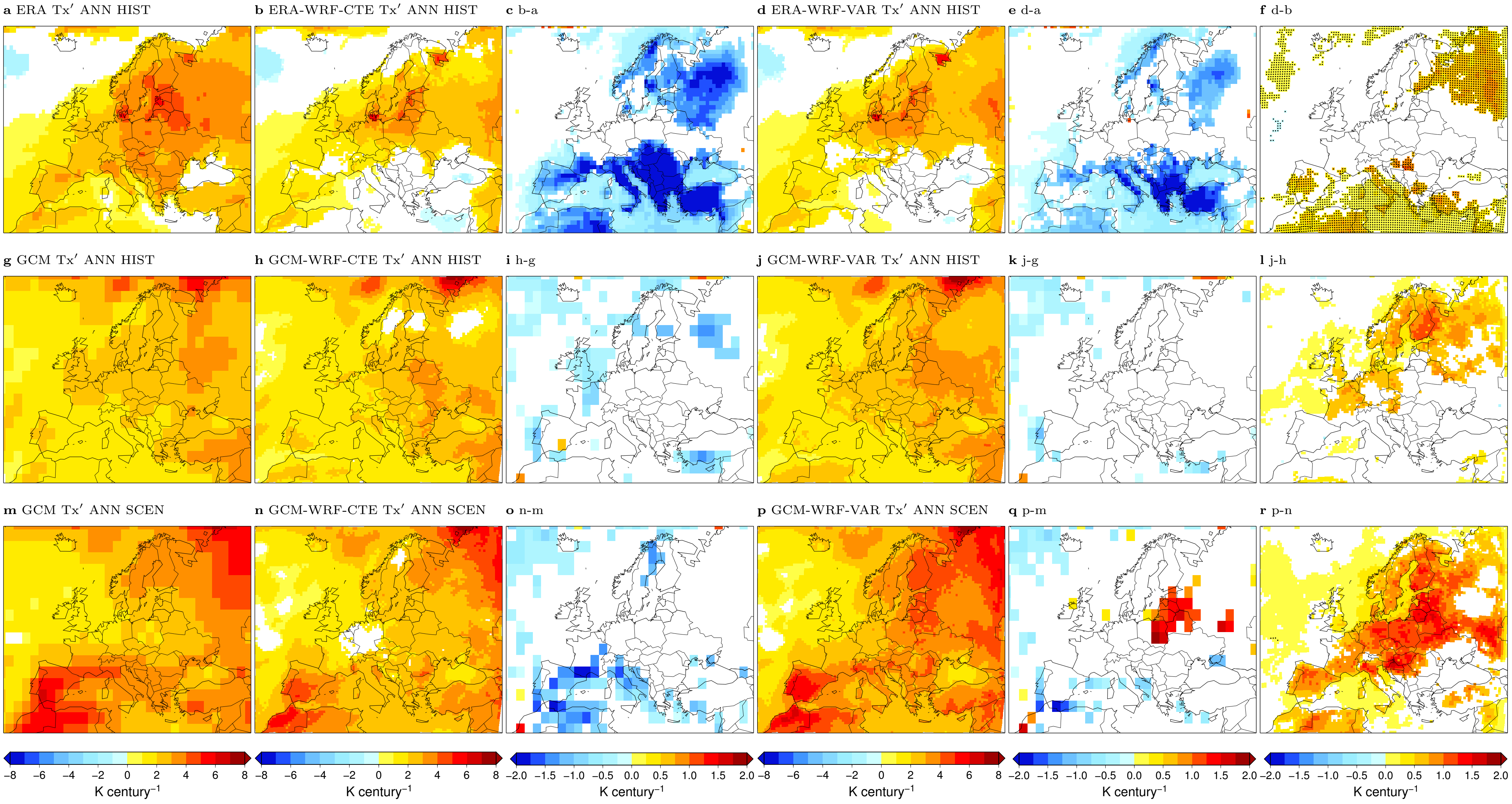

Supplementary Figure 4: As Supp. Fig. 1 for Tx.

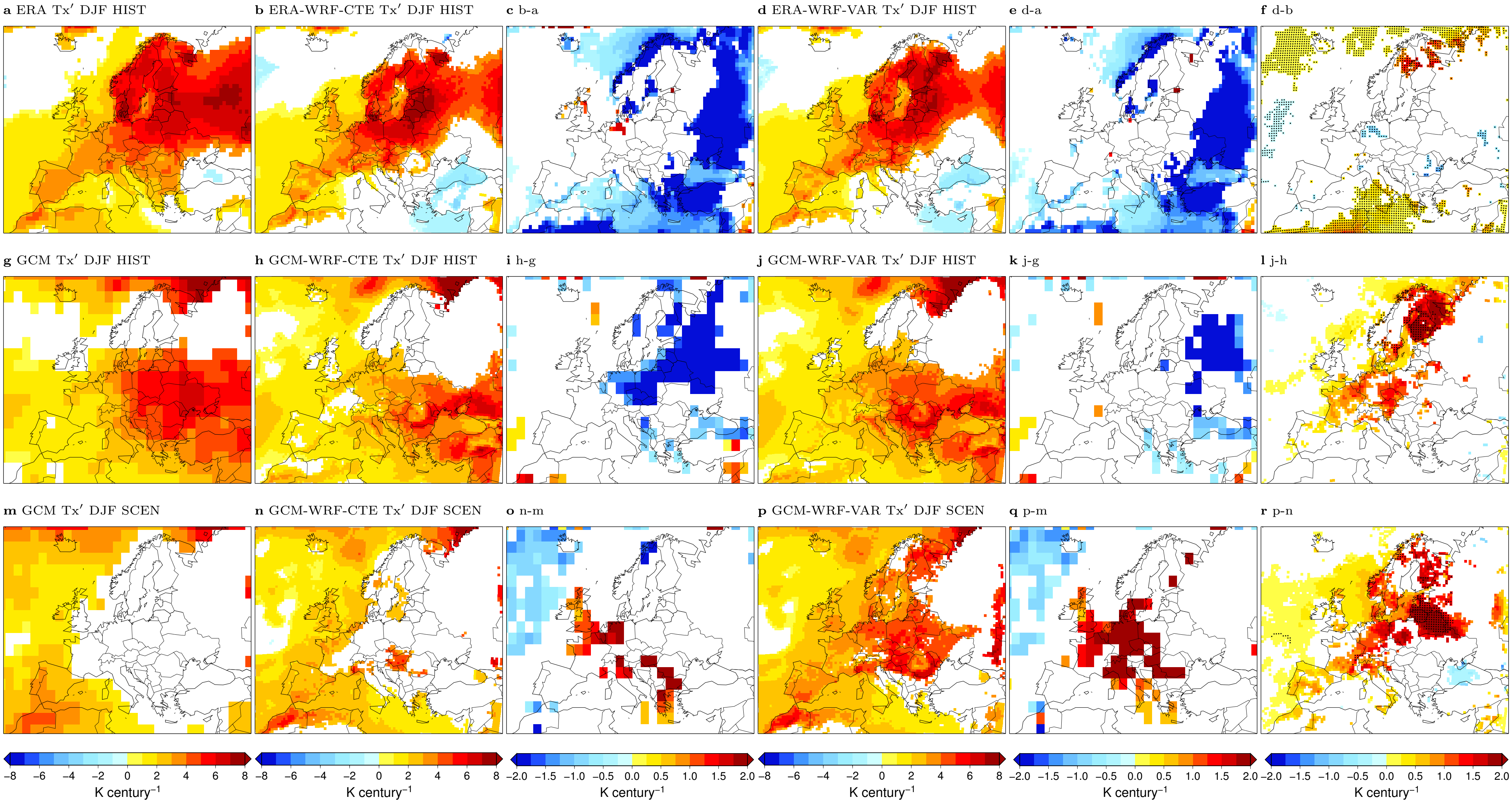

Supplementary Figure 5: As Supp. Fig. 1 for DJF-mean Tx.

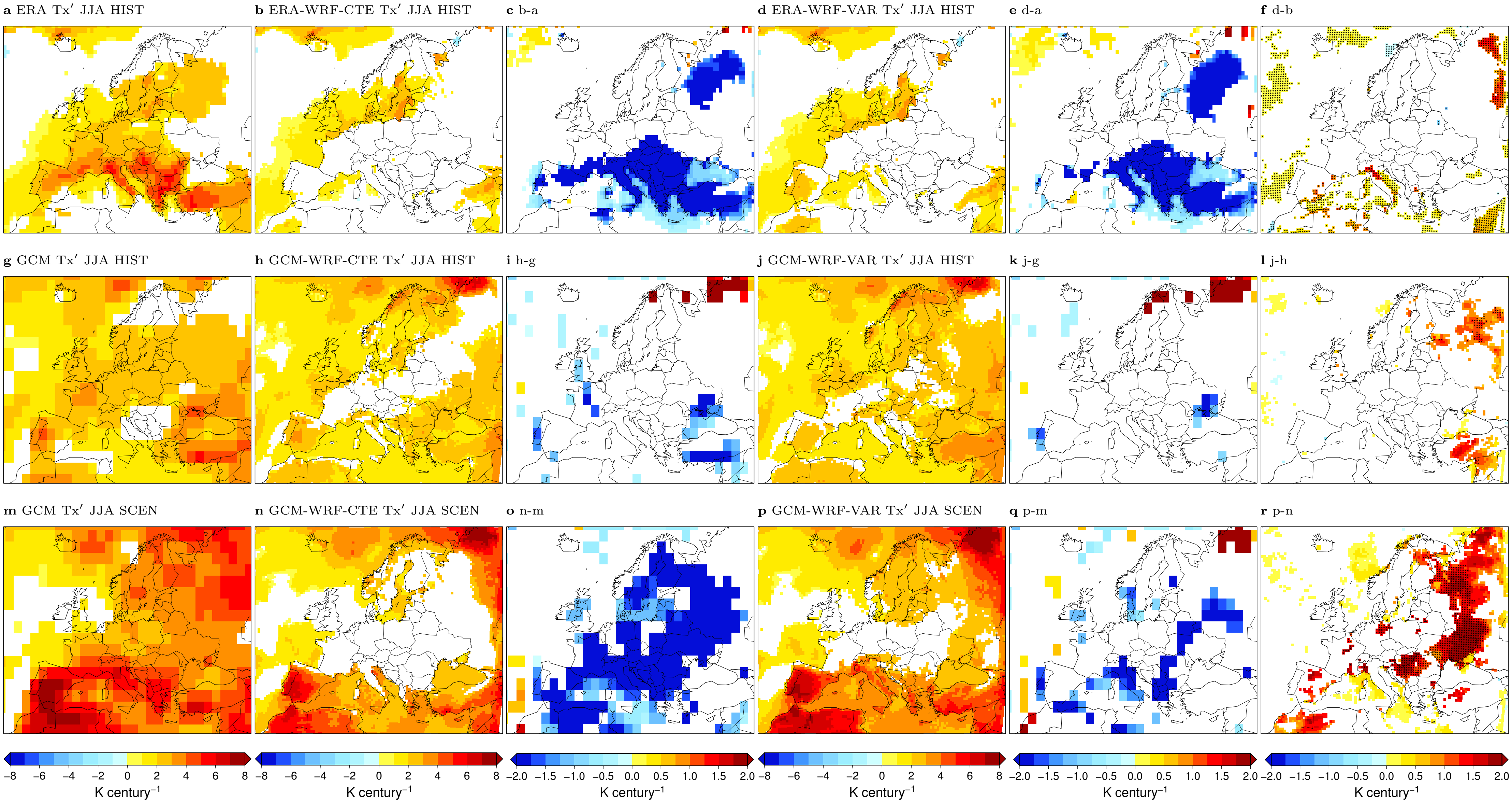

Supplementary Figure 6: As Supp. Fig. 1 for JJA-mean Tx.

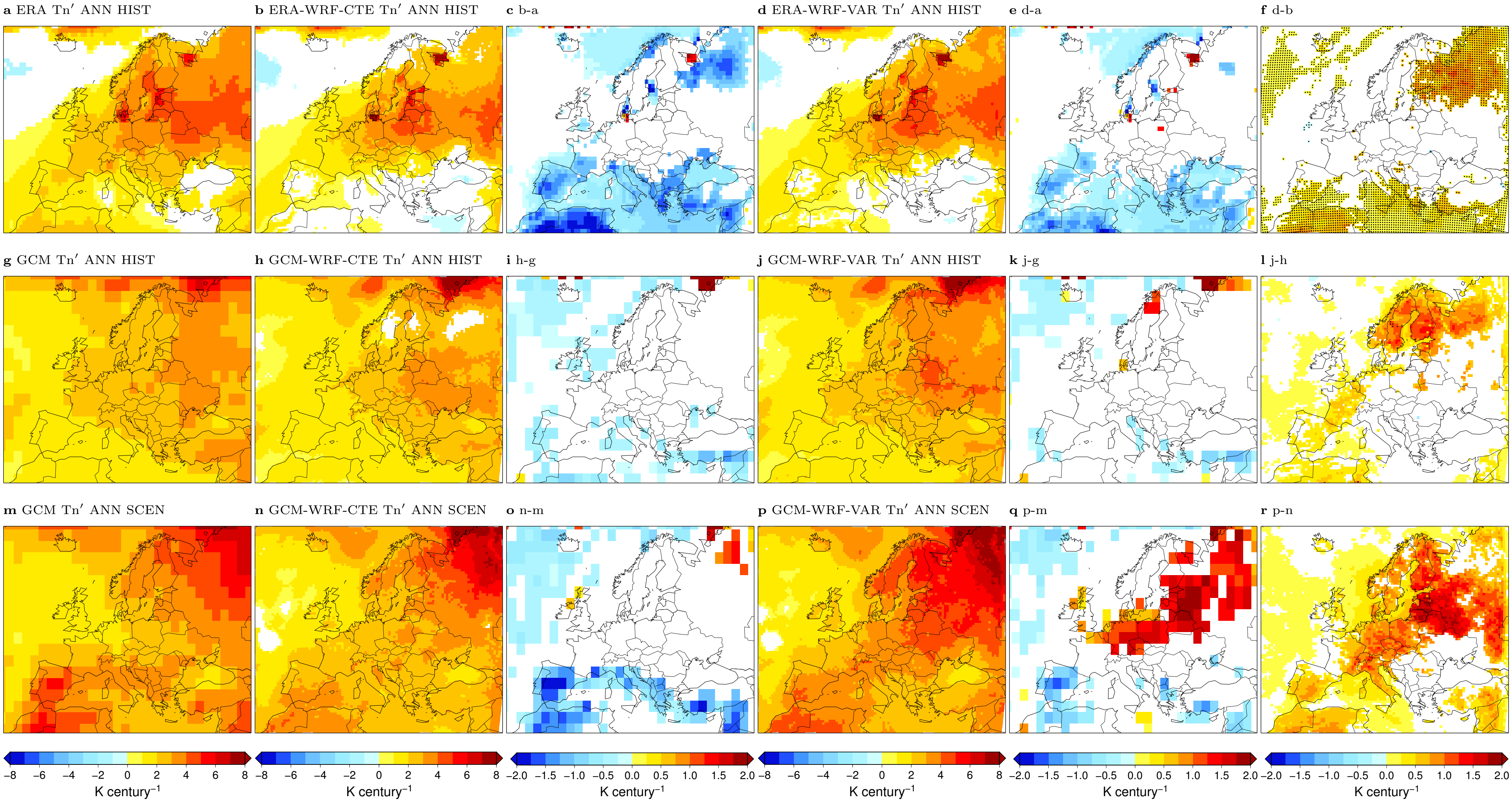

Supplementary Figure 7: As Supp. Fig. 1 for Tn.

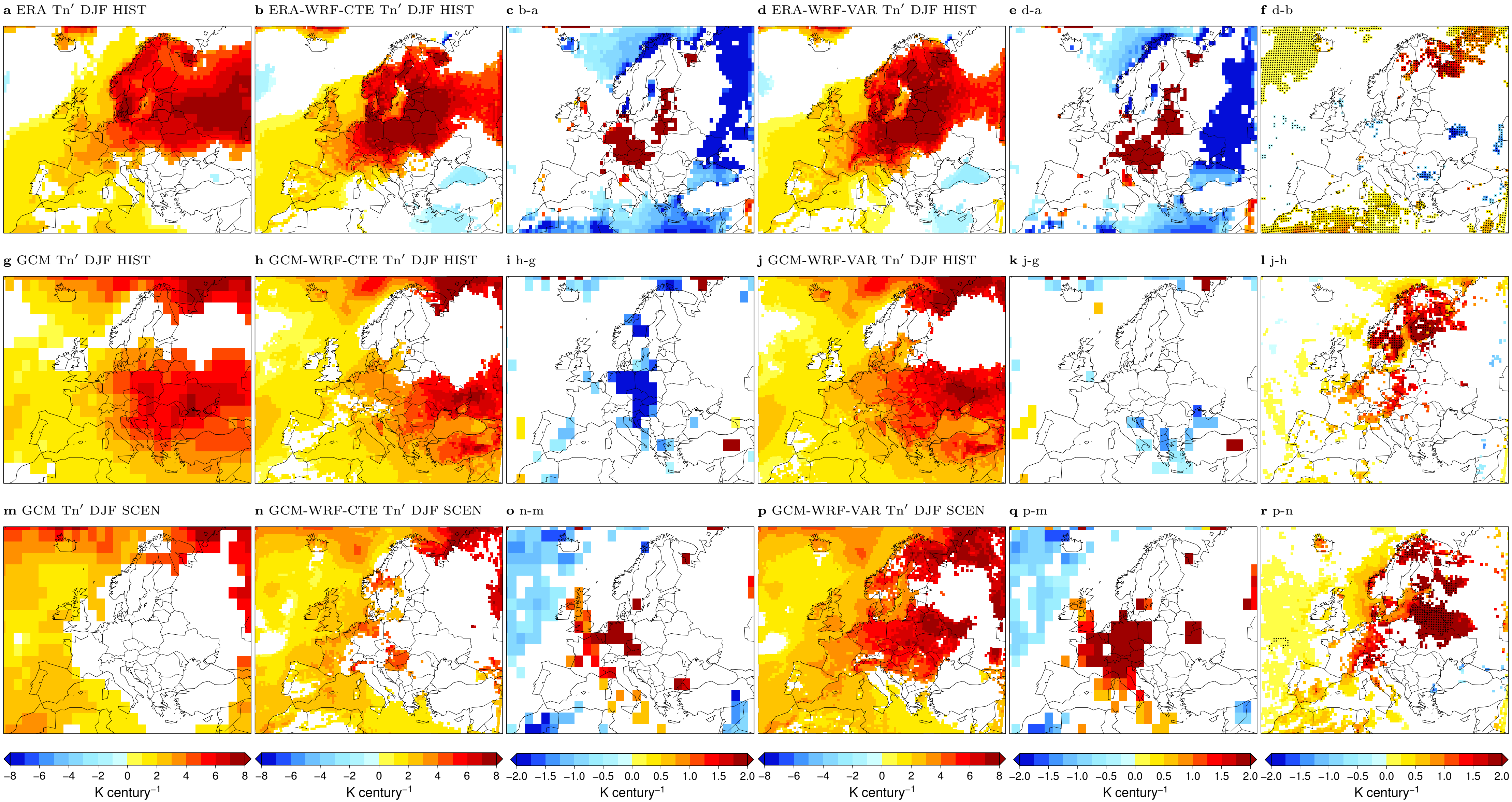

Supplementary Figure 8: As Supp. Fig. 1 for DJF-mean Tn.

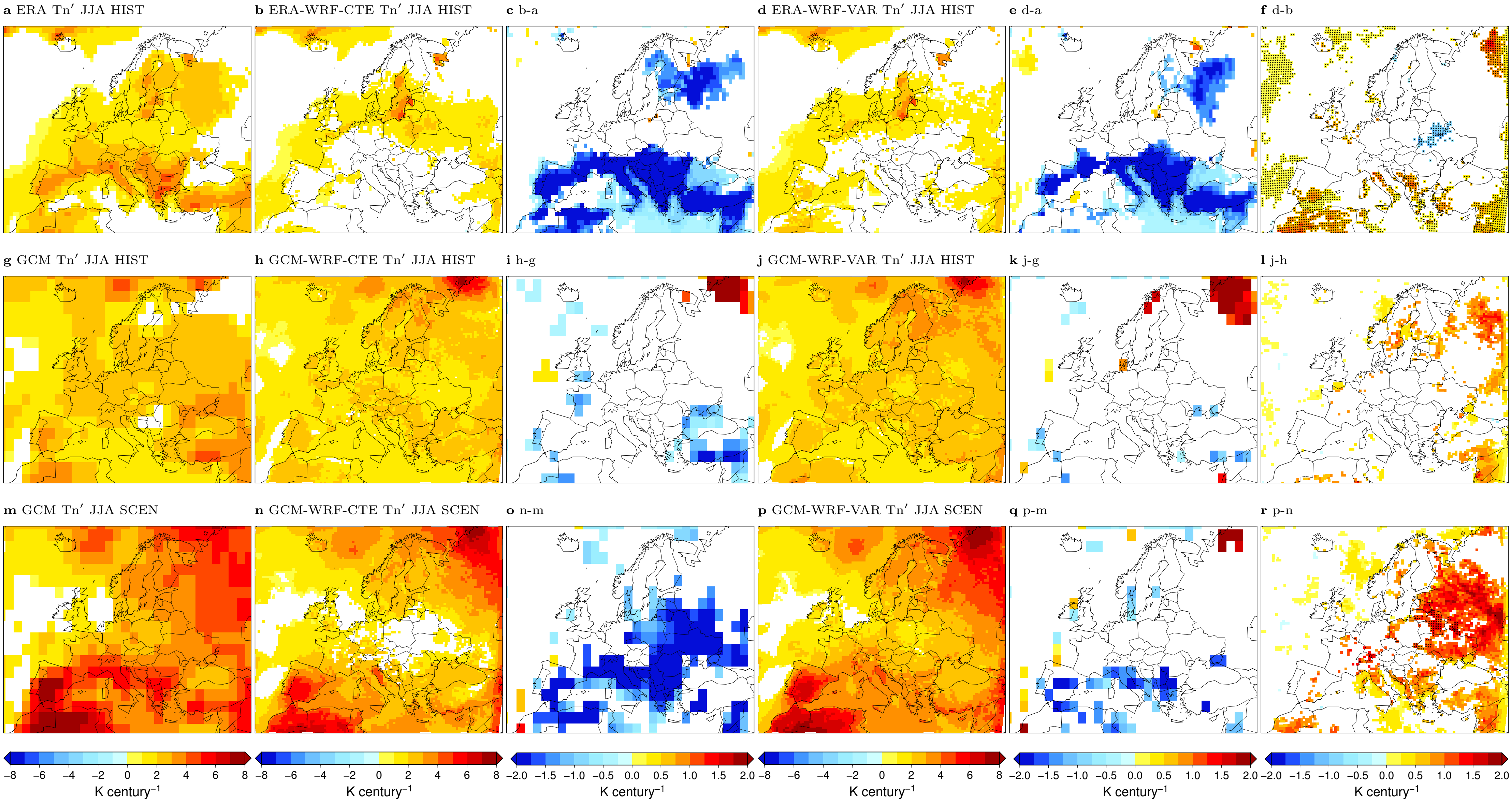

Supplementary Figure 9: As Supp. Fig. 1 for JJA-mean Tn.

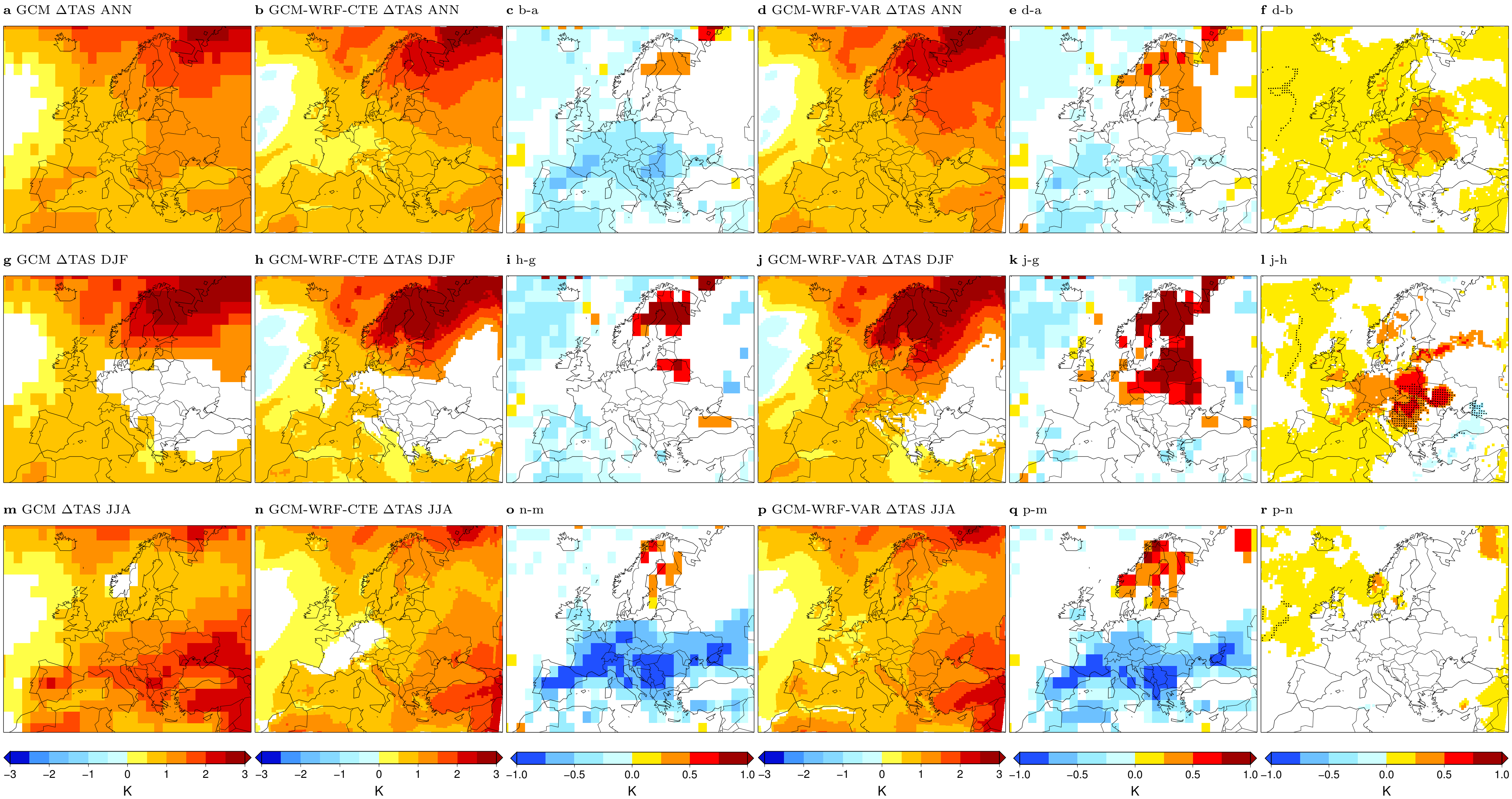

Supplementary Figure 10: Projected changes (2011-2040 vs. 1971-2000 climatologies, i.e. under 1.5^{o}C global warming) in year-mean (first row), DJF-mean (second row) and JJA-mean (third row) TAS from GCM (first column) and GCM-driven WRF experiments (CTRL configuration, see Table 1 in the main manuscript), CTE and VAR (second and fourth columns, respectively), along with the differences between ones and others: CTE minus GCM (c), VAR minus GCM (e) and VAR minus CTE (f). Only significant values ($p<0.1$) are shown. The points in the last column indicate that the magnitude of the difference between the VAR and CTE experiments is equal to or greater than the magnitude of the signal from the respective CTE experiment. Units: K. (Note: this is Figure 2 in the article.)

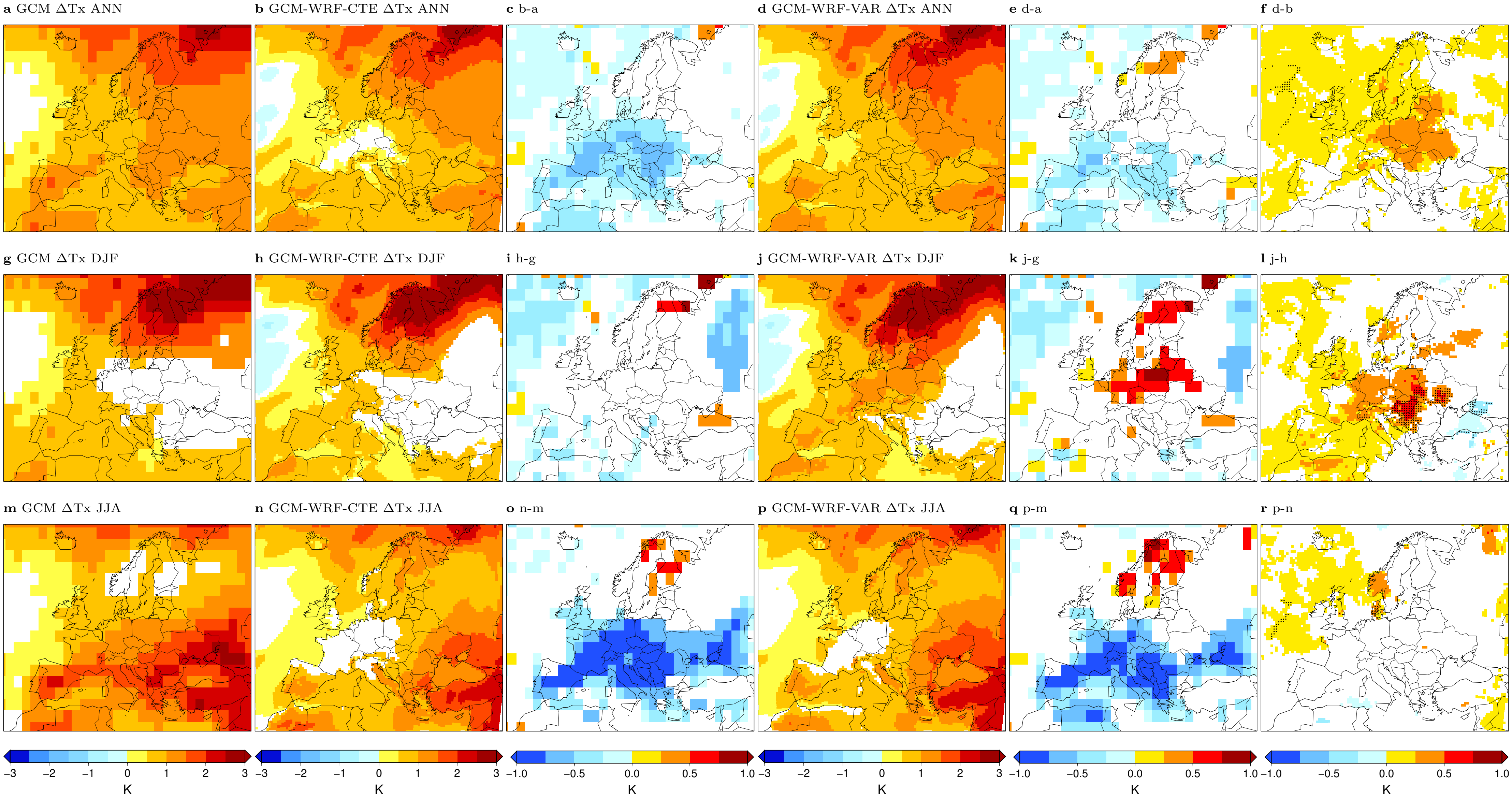

Supplementary Figure 11: As Supp. Fig. 10 for Tx.

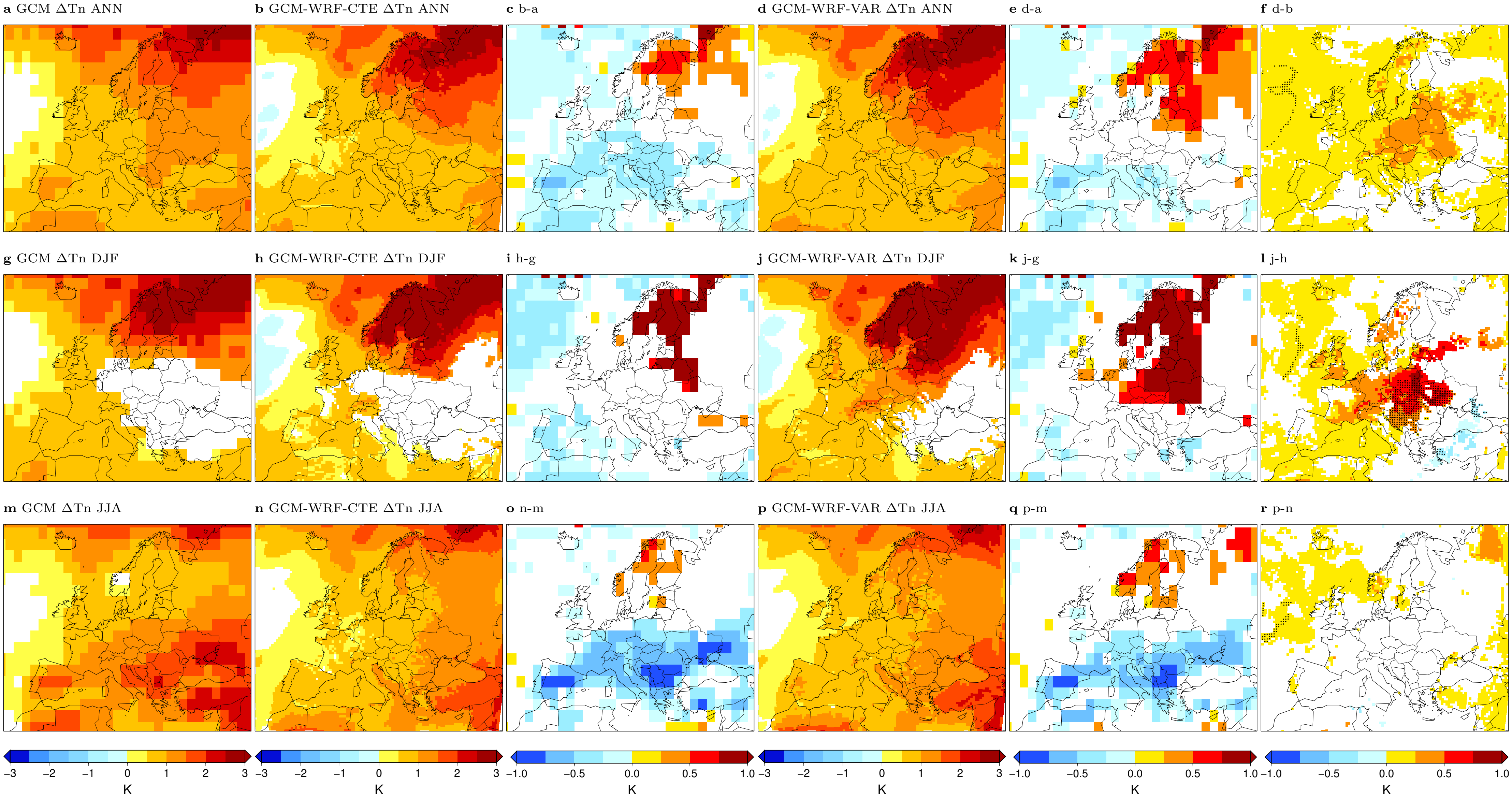

Supplementary Figure 12: As Supp. Fig. 10 for Tn.

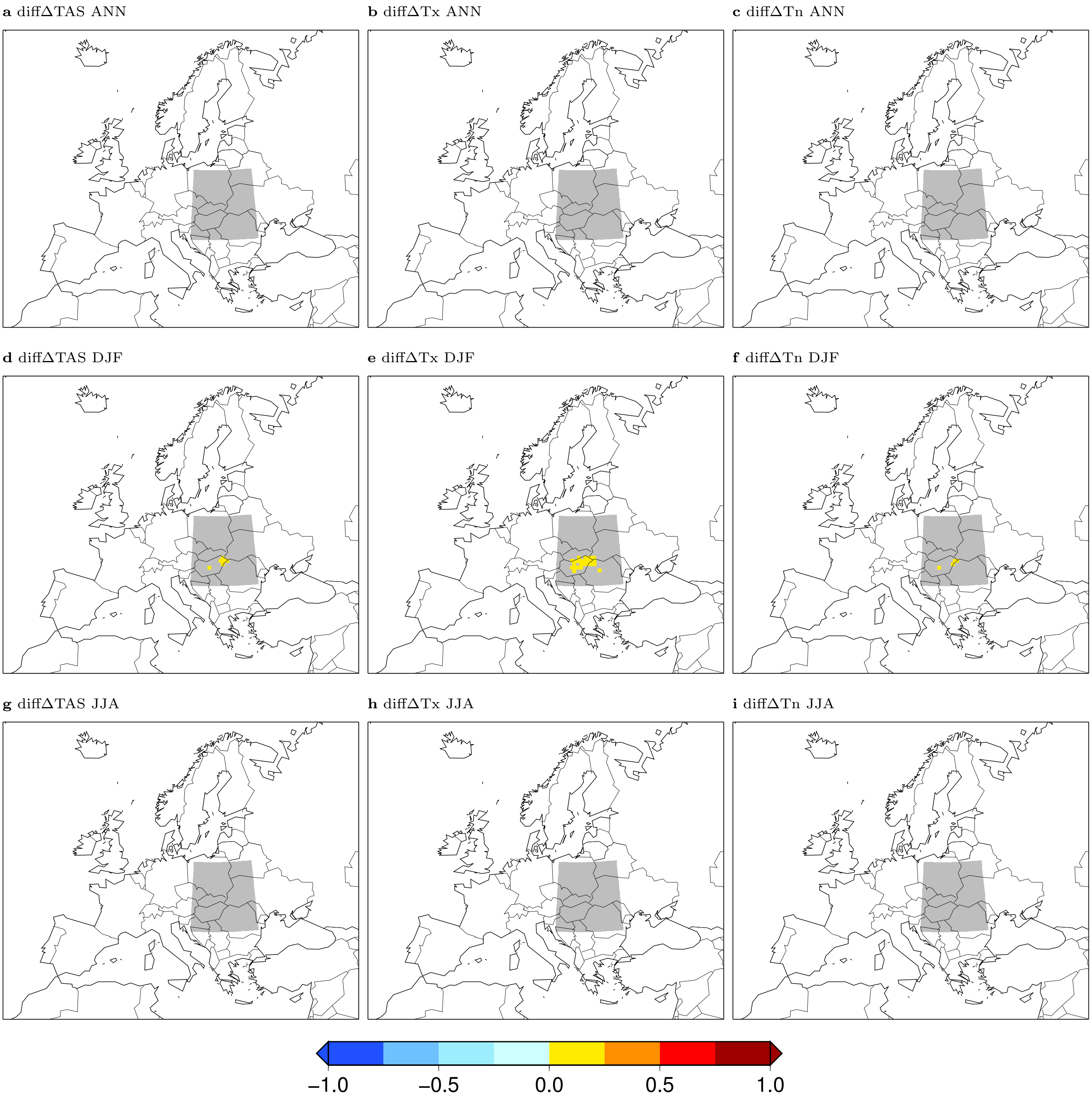

Supplementary Figure 13: Differences in the projections (under 1.5^{o}C global warming) for TAS, Tx and Tn (first, second and third column respectively; annual, DJF and JJA averages in first, second and third rows respectively) between approaches (VAR minus CTE) from the experiments (CTRL configuration) run over the small domain shaded in gray instead of over the whole Euro-Cordex domain. Only significant values ($p<0.1$) are shown. Units: K. Note that the impact of the domain size on the senitivity of these projections to the varying GHG approach can be inferred by comparison with the last columns of Supp. Figs. 10, 11 and 12. The absence of points in these plots indicate that the magnitude of the difference between the VAR and CTE experiments never is equal to or greater than the magnitude of the signal from the respective CTE experiment in the small domain experiments.

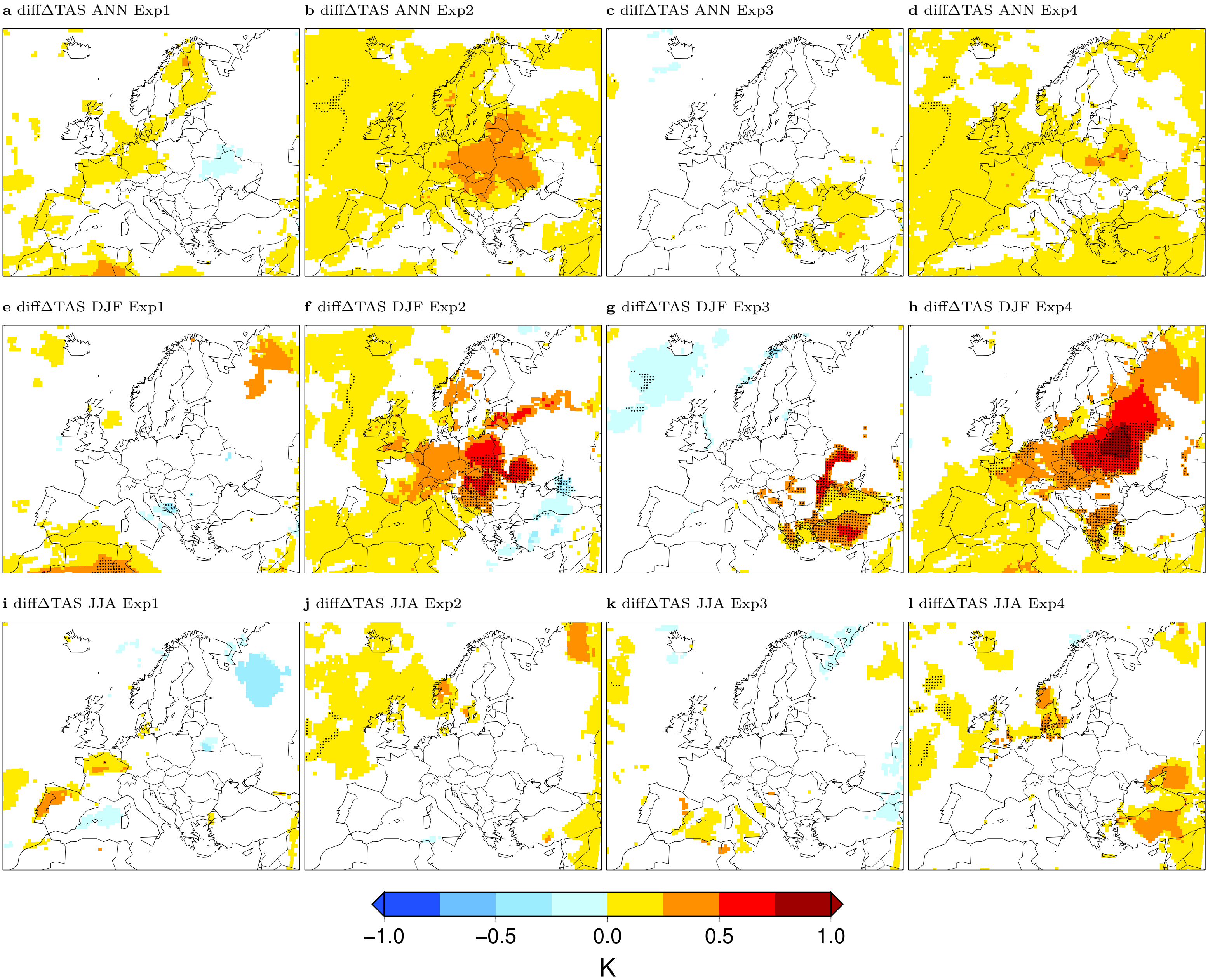

Supplementary Figure 14: Differences in the projections for TAS (annual, DJF and JJA averages in first, second and third rows, respectively) between approaches (VAR minus CTE) from each experiment of the ensemble (see Table 1 in the main manuscript). The points indicate that the magnitude of the difference between the VAR and CTE experiments is equal to or greater than the magnitude of the signal from the respective CTE experiment. Only significant values ($p<0.1$) are shown. Units: K. (Note that second column here is the sixth column of Supp. Fig. 10.)

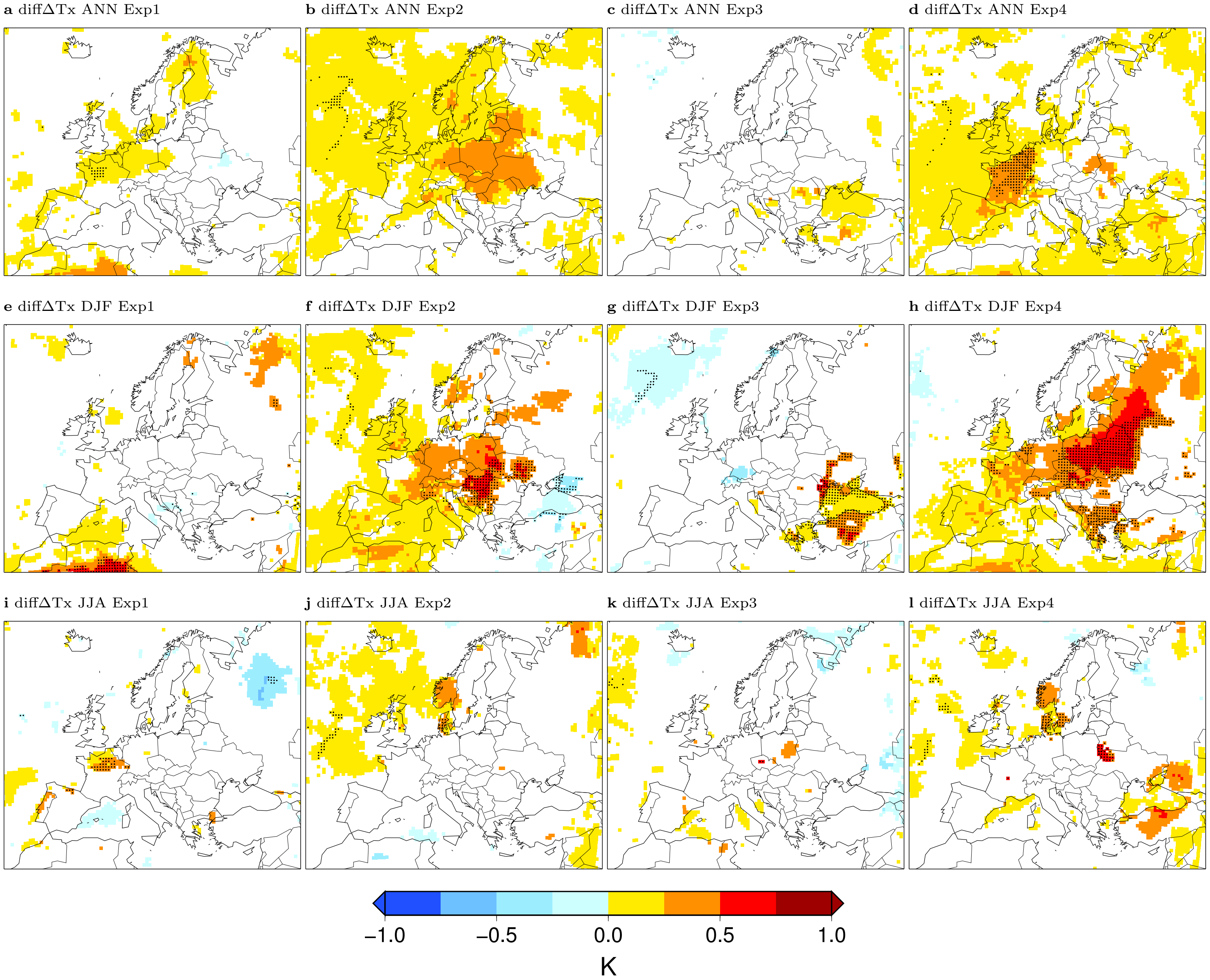

Supplementary Figure 15: As Supp. Fig. 14 for Tx. (Note that second column here is the sixth column of Supp. Fig. 11.)

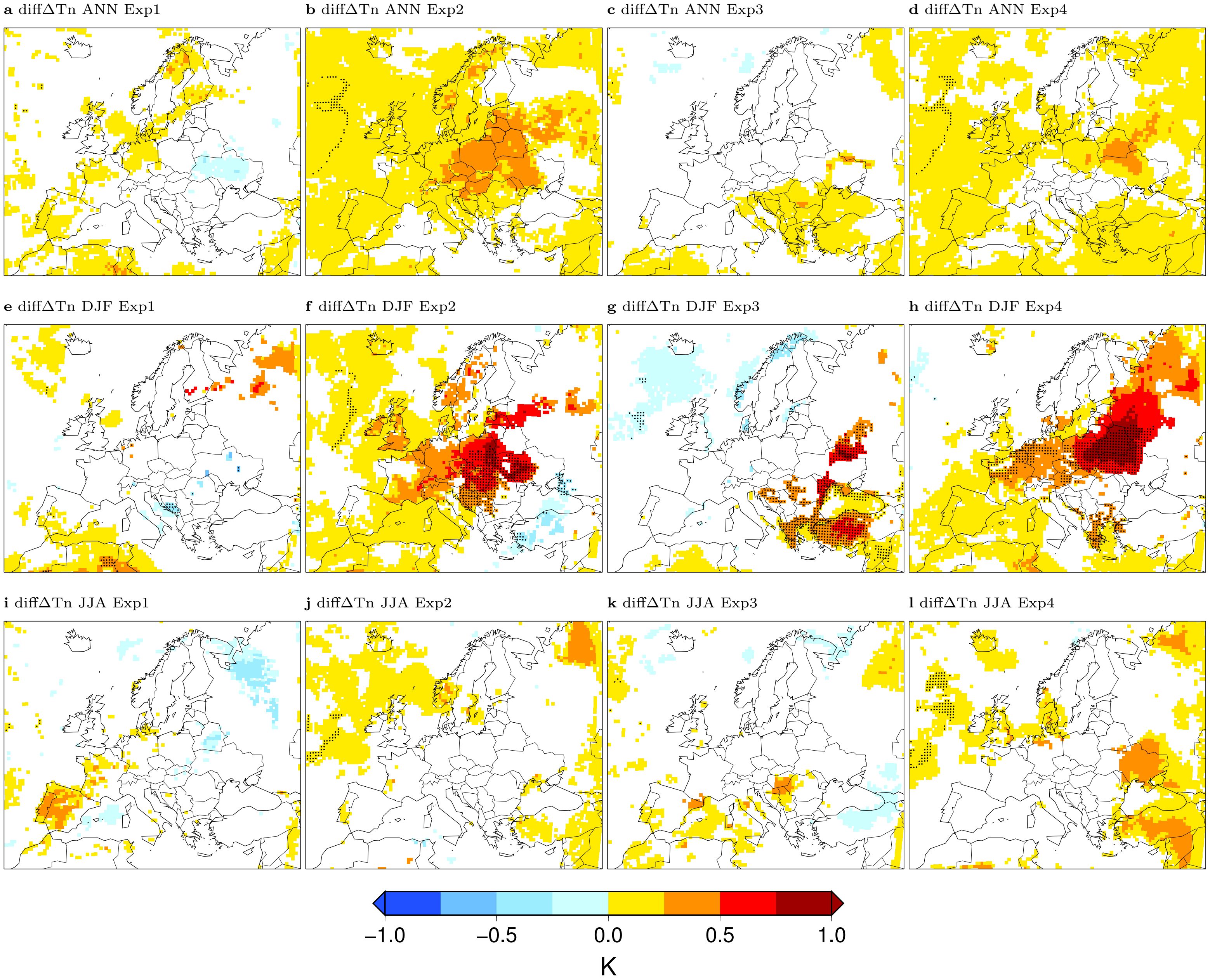

Supplementary Figure 16: As Supp. Fig. 14 for Tn. (Note that second column here is the sixth column of Supp. Fig. 12.)

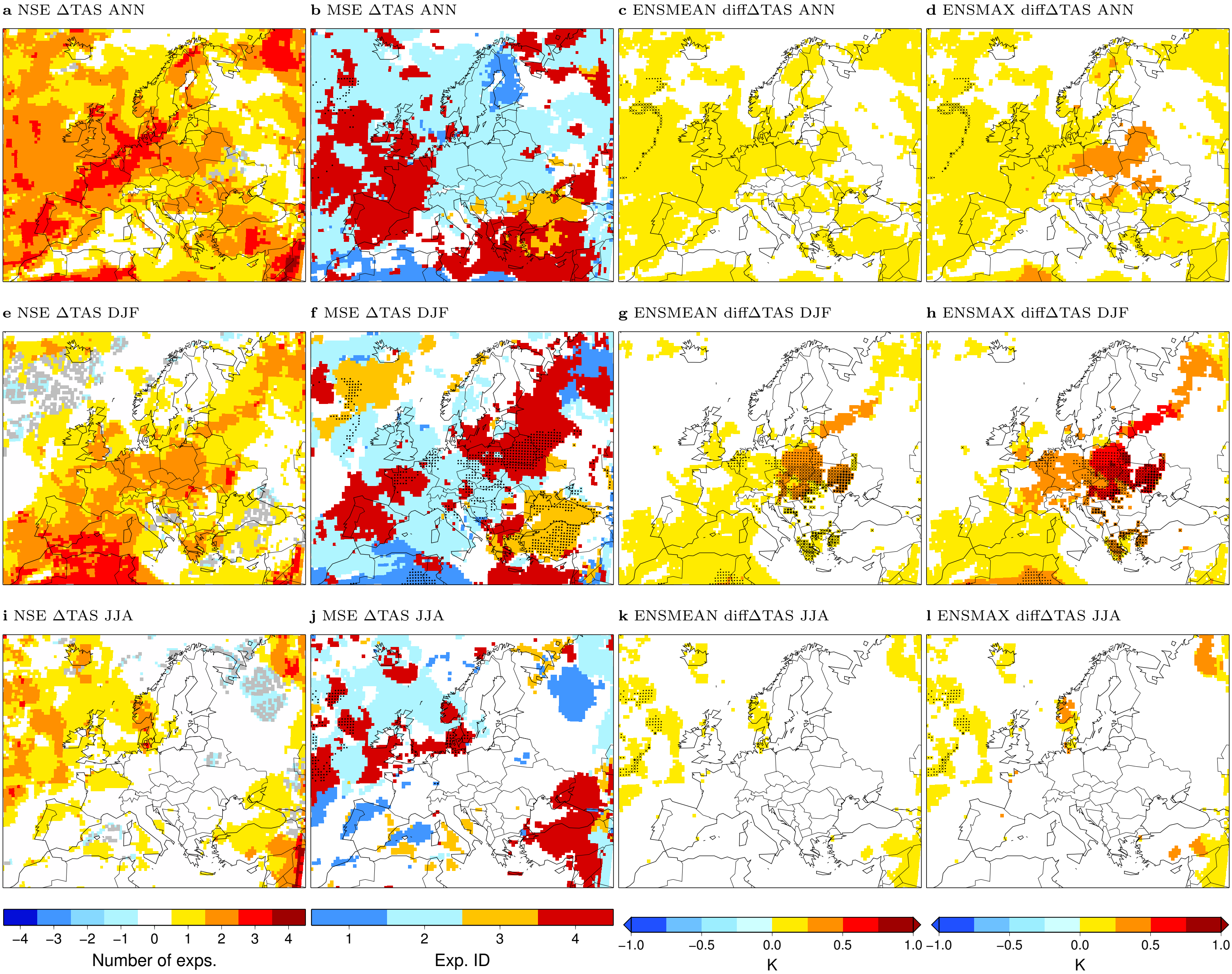

Supplementary Figure 17: Number of sensitive experiments (NSE, first column) and most sensitive experiment (MSE, second column, numbers referred to the experiments identifiers of Table 1 in the main manuscript), within the ensemble of WRF runs (see Table 1 in the main manuscript), to the GHG handling by the RCM when projecting TAS changes under 1.5^{o}C global warming (ΔTAS, assessed at annual, DJF and JJA scales in first, second and third rows, respectively). The NSE is computed by assigning 1 to those experiments in which the varying GHG approach provides significantly ($p<0.1$) higher values of ΔTAS than the constant GHG approach, -1 to those in which ΔTAS is significantly lower in the varying GHG counterpart of each experiment, 0 if there is no significant difference between both approaches, and then summing over the four experiments; with the color gray indicating the mix of 1 and -1. MSE denotes the experiment in which the difference in ΔTAS between approaches is highest if it is statistically significant; if not, it is white. The points in the second column indicate that ΔTAS in the MSE is at least double when GHG vary. The third and fourth columns depict the ensemble mean and maximum values of the differences in ΔTAS (units: K) between approaches over the grid points in which at least two experiments provide significant values of such differences; otherwise, it is white. The points, crosses, stars and squares in the third and fourth columns indicate that ΔTAS is at least double when GHG vary in one, two, three or four experiments, respectively. (Note: this is Figure 3 in the article.)

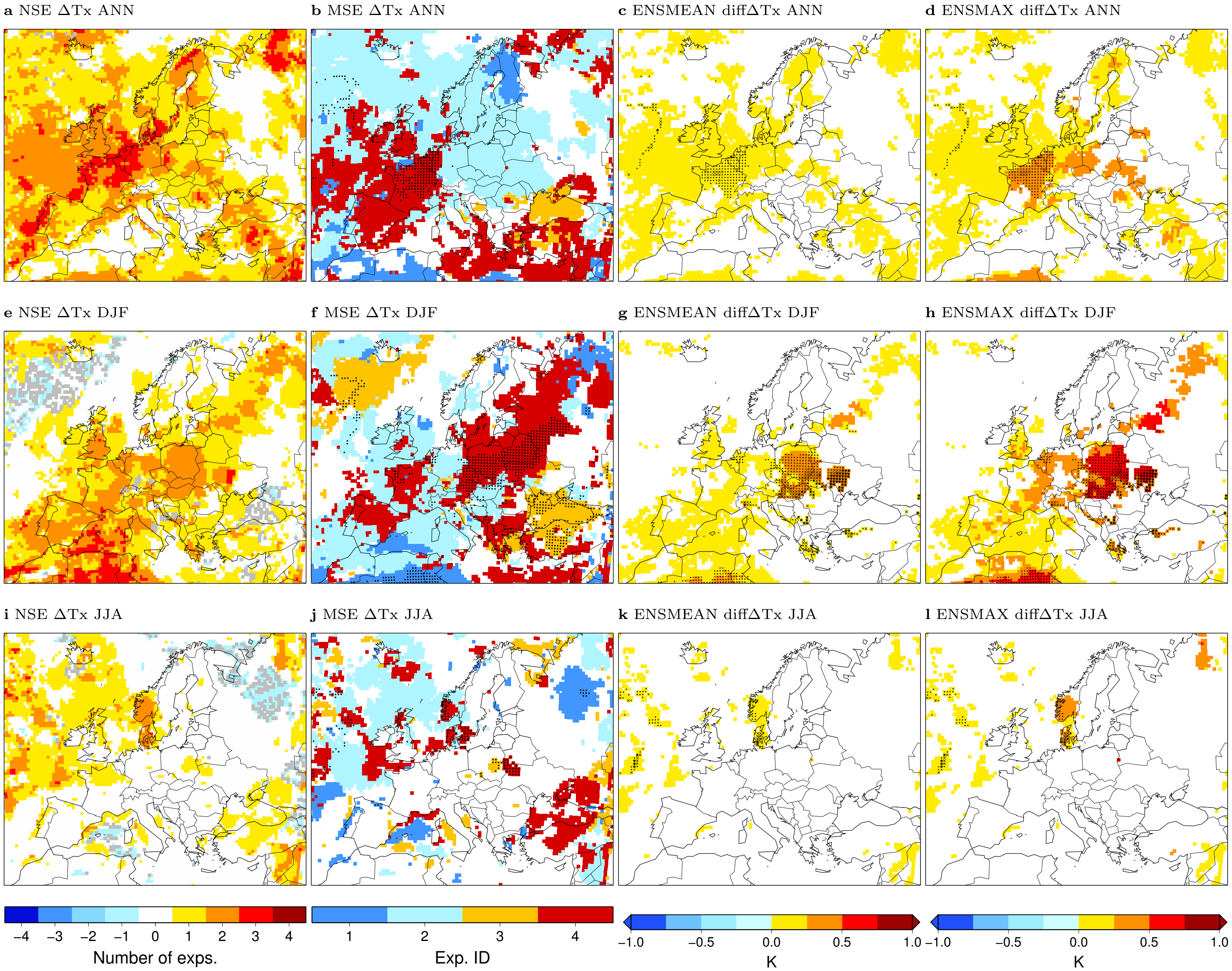

Supplementary Figure 18: As Supp. Fig. 17 for Tx.

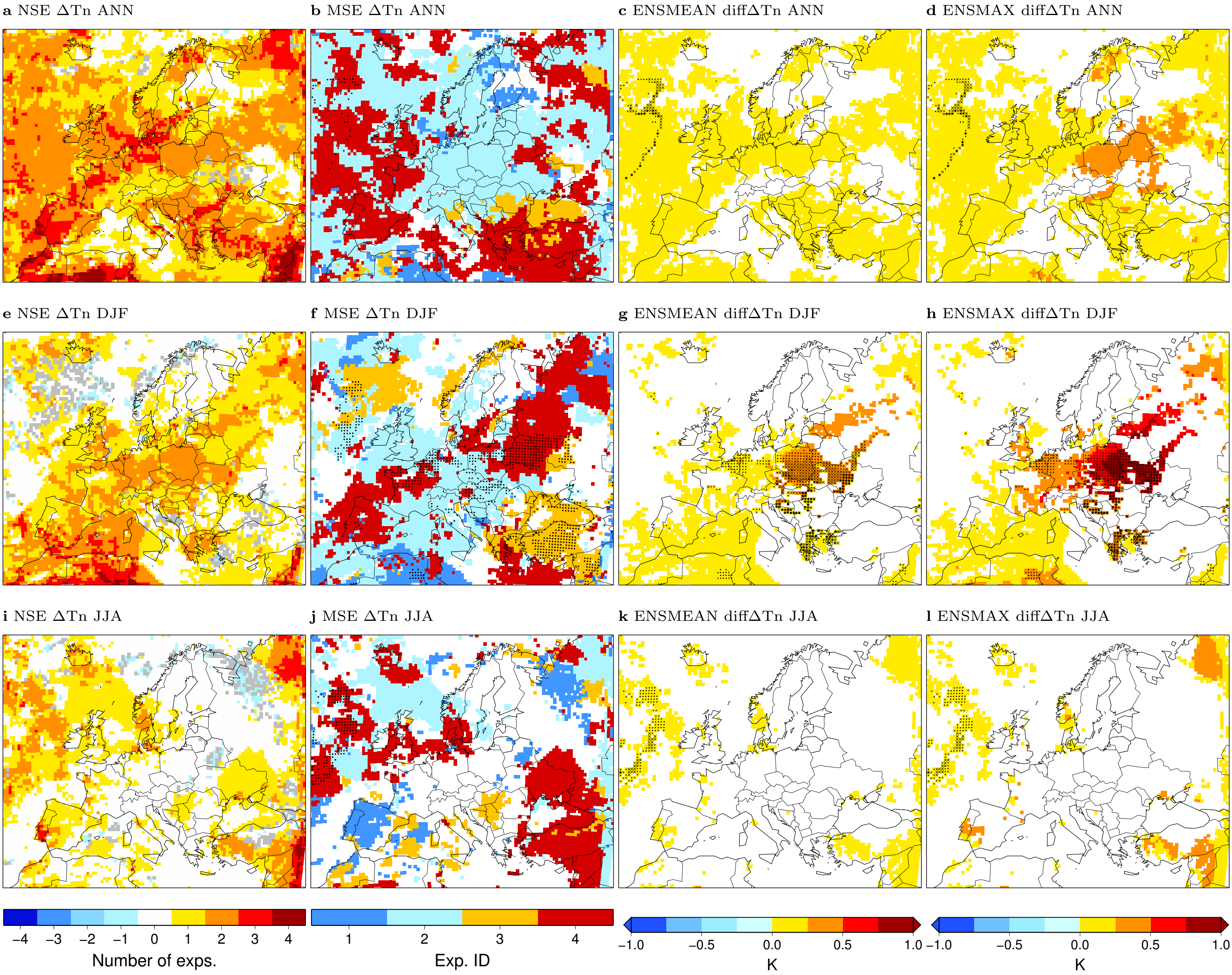

Supplementary Figure 19: As Supp. Fig. 17 for Tn.

Supplementary Table 1: Details of the WRF namelist.wps used for the setup of the regional simulations.

```
&share
 wrf_core = 'ARW',
 max_dom = 1,
 start_date = '2001-09-01-00_00:00:00',
 end_date = '2006-01-01-00_00:00:00',
 interval_seconds = 21600,
 io_form_geogrid = 2,
 opt_output_from_geogrid_path = '.',
 debug_level = 0,
/

&geogrid
 parent_id = 1,
 parent_grid_ratio = 1,
 i_parent_start = 1,
 j_parent_start = 1,
 e_we = 120,
 e_sn = 117,
 s_we = 1,
 s_sn = 1,
 geog_data_res = '10m',
 dx = 0.44,
 dy = 0.44,
 map_proj = 'lat-lon',
 ref_lat = 60.21,
 ref_lon = -44.14,
 truelat1 = 60.0,
 truelat2 = 30.0,
 stand_lon = -18.0,
 ref_x = 8,
 ref_y = 110,
 pole_lon = -162,
 pole_lat = 39.25,
 geog_data_path = '.'
 opt_geogrid_tbl_path = '.',
/

&ungrib
 out_format = 'WPS',
 prefix = 'FILE',
/

&metgrid
 fg_name = 'FILE', 'PRES'
 io_form_metgrid = 2,
 opt_output_from_metgrid_path = '.',
 opt_metgrid_tbl_path = '.',
/
```

```
&mod_levs
 press_pa = 201300 , 200100 , 100000 ,
             95000 ,  90000 ,
             85000 ,  80000 ,
             75000 ,  70000 ,
             65000 ,  60000 ,
             55000 ,  50000 ,
             45000 ,  40000 ,
             35000 ,  30000 ,
             25000 ,  20000 ,
             15000 ,  10000 ,
              5000 ,   1000
/
```

Supplementary Table 2: Details of the WRF namelist.input used for the setup of the regional simulations.

```
&time_control
 start_year                  = 2001,
 start_month                 = 09,
 start_day                   = 01,
 start_hour                  = 00,
 start_minute                = 00,
 start_second                = 00,
 end_year                    = 2006,
 end_month                   = 01,
 end_day                     = 01,
 end_hour                    = 00,
 end_minute                  = 00,
 end_second                  = 00,
 interval_seconds            = 21600
 input_from_file             = .true.,
 history_interval            = 60,
 frames_per_outfile          = 240,
 restart                     = .false.,
 restart_interval            = 1440,
 write_hist_at_0h_rst        = .true.,
 auxinput4_inname            = "wrflowinp_d<domain>"
 auxinput4_interval          = 360,
 io_form_history             = 2
 io_form_restart             = 2
 io_form_input               = 2
 io_form_boundary            = 2
 debug_level                 = 0
 io_form_auxinput4           = 2
/

&domains
 time_step                   = 300,
 time_step_fract_num         = 0,
 time_step_fract_den         = 1,
 max_dom                     = 1,
 e_we                        = 120,
 e_sn                        = 117,
 e_vert                      = 29,
 p_top_requested             = 5000,
 num_metgrid_levels          = 48,
 num_metgrid_soil_levels     = 5,
 dx                          = 48918.09,
 dy                          = 48918.09,
 grid_id                     = 1,
 parent_id                   = 1,
 i_parent_start              = 1,
 j_parent_start              = 1,
 parent_grid_ratio           = 1,
 parent_time_step_ratio      = 1,
```

```
 feedback                   = 1,
 smooth_option              = 2,
 max_ts_locs                = 8,
 sfcp_to_sfcp               = .true.,
 use_surface                = .false.,
/

&physics
 progn                      = 1,
 mp_physics                 = 2, ← for Lin et al. Scheme
#mp_physics                 = 10, ← for Morrison 2-moment Scheme
 ra_lw_physics              = 4, ← for RRTMG Longwave Scheme
#ra_lw_physics              = 3, ← for CAM Longwave Scheme
 ra_sw_physics              = 4, ← for RRTMG Shortwave Scheme
#ra_sw_physics              = 3, ← for CAM Shortwave Scheme
 radt                       = 50,
 sf_sfclay_physics          = 1,
 sf_surface_physics         = 2,
 bl_pbl_physics             = 1,
 bldt                       = 0,
 cu_physics                 = 5, ← for Grell 3D Ensemble Scheme
#cu_physics                 = 1, ← for Kain-Fritsch Scheme
 cu_diag                    = 1,
 cudt                       = 0,
 isfflx                     = 1,
 ifsnow                     = 1,
 icloud                     = 1,
 surface_input_source       = 1,
 num_soil_layers            = 4,
 sf_urban_physics           = 0,
 topo_wind                  = 1,
 sst_update                 = 1,
 cu_rad_feedback            = .true.,
/

&dynamics
 w_damping                  = 1,
 diff_opt                   = 1,
 km_opt                     = 4,
 diff_6th_opt               = 0,
 diff_6th_factor            = 0.12,
 base_temp                  = 290.
 damp_opt                   = 0,
 zdamp                      = 5000.,
 dampcoef                   = 0.2,
 khdif                      = 0,
 kvdif                      = 0,
 non_hydrostatic            = .true.,
 moist_adv_opt              = 2,
 scalar_adv_opt             = 2,
 chem_adv_opt               = 0,
```

```
 tke_adv_opt                    = 2,
 time_step_sound                = 4,
 h_mom_adv_order                = 5,
 v_mom_adv_order                = 3,
 h_sca_adv_order                = 5,
 v_sca_adv_order                = 3,
/

&bdy_control
 spec_bdy_width                 = 5,
 spec_zone                      = 1,
 relax_zone                     = 4,
 specified                      = .true.,
 nested                         = .false.,
/

&namelist_quilt
 nio_tasks_per_group            = 0,
 nio_groups                     = 1,
/
```
